# Modern Sociology

**Published:** 1897-01-30

**Authors:** 


### Modern Sociology.

### PAUPERS AND CRIMINALS IN NEW YORK.

Until the beginning of last year (1896) the paupers, the State-supported sick and insane, and the criminals of New York were under the management of one department, known as the Department of Charities and Correction. When a city is small it is practical, perhaps, for all its dependants, however great be the difference in their merits, to be looked after by one board, but when any class of these becomes numerous confusion and abuses are sure to arise. This was the case in New York city. There was but one purse for all the different classes of wards of the city, and therefore it was thought right and economical—probably right because economical—to utilise the services of prisoners as attendants on the sick and the insane. The results might be guessed. The thieves proved worthy of their reputation, and stole from their patients any little valuables they possessed. In one case an insane young girl had her hair, which was long and beautiful, cut off by her robber attendant, who meant to sell it to a wig-maker. It is more than forty years since wise philanthropists began to agitate for a change in these arrangements, and were rewarded by getting rid of criminals for nurses. But in the laundry, kitchen, &c., of various charitable institutions they lingered till as late as October 1st, 1895. But it was found that, under competent paid supervision, lunatics could be trusted to do much of the institution work which had formerly fallen to prisoners, and in the hospitals the creation of nurse-training schools did away with any possible need for their services.

It is not to be supposed, however, that the results of these past abuses are quite overcome. In a report of twenty years ago we read the following: "The pauper children at Randall's Island, twelve hundred in number, are placed in daily contact with the most abandoned women of the city, women convicted in the police-courts of drunkenness and debauchery, and sentenced to the workhouse for six and three months." It will take more than twenty years for the evil effects of this association to pass away from society. The mere fact that New York is the Venice of the New World, and that the various charitable and penal institutions are situated on different islands, threw the criminal and the unfortunate together. Together they shivered or scorched on the pier waiting for the steamer that was to take them to their several destinations, and at every turn of their career there was something to give the impression that poverty and vice were equally criminal in the eyes of the city of New York.

It is not to be pretended that the separation of the Departments of Charities and Correction from each other has been accomplished without inconvenience. Take, for example, the case of affairs at the Metropolitan Hospital. "The erection of a fire-escape, begun by convicts from the penitentiary in November, 1895, is a good illustration of the dangers attending the use of convict labour. The work was interrupted on January 1st by the doubt as to the legality of utilising penitentiary labour in this manner, under the Act dividing the departments. The Act was amended so as to expressly authorise such use of penitentiary men, the amended Bill becoming law on March 11th. The work was not resumed, however, for some time, and was finally undertaken by workhouse men. At this date (October 1st, 1896) the fire-escape is still unfinished. Scores of lives have thus been kept in peril for nearly a year." In a subsequent paragraph the report from which we are quoting says: "It is ascertained from the report of a visit to the Metropolitan Hospital on October 15th, that work on the fire-escape has again come to a standstill. The supply of material is said to have given out. Meanwhile a large hole has been broken through the wall into the women's ward, on the first floor, extending from the window-sill to the floor, and of the width of the window. This hole is closed only by a rubber cloth. Another opening, at which a second entrance has been broken, also admits a good deal of cold air. There are twenty beds in this ward. To the danger from fire is thus added the danger arising from copious draughts of cold air." Alas, poor fire-escape! This is almost worthy of Dickens's circumlocution office!

On the other hand, the free hand now given to the Department of Charities has enabled much-needed improvements and additions to be made to some hospitals. In Gouverneur Hospital, for example, a year ago there were no bath-rooms, no hot water above the basement except in the operating-room, and no means of disinfecting clothing. The room in which post-mortem examinations were held was a mere closet off the hall. Worst of all, the nurses' and physicians' dining-room was a part of the operating-room, the partition between them reaching only part of the way to the ceiling. Most of these defects have been remedied during the past year.

It is evident, however, that much remains to be done. The almshouse, the home of the infirm poor, is thus reported on: "The almshouse is continuously and excessively overcrowded. During the past summer more than three hundred patients slept on beds on the floor, and in winter the number has been much larger. Fifteen hundred inmates occupy buildings four storeys high, which have neither hot nor cold water, no bathrooms, no lavatories, and only outside, uncovered stairs. The food is never served hot, and lacks variety, the cost of food for the inmates of the almshouse proper being between five and six cents per day. In the almshouse hospital there is but one nurse (untrained) for every forty patients." A subsequent note says: "The proper capacity of the almshouse is 1,800. The highest number of inmates at one time was 2,962, on which date the inmates slept, three in two beds, throughout the main buildings, and there were also 530 sleeping on the floor, 48 in tents, 15 in the waiting-room, 43 in a basement, and 160 in the smoking-shed."

The condition of things here seems as bad as it well can be. In the City Hospital there are also serious defects, but as far as mere repairs go things are being improved. But new buildings seem to be needed for various special diseases. In the epileptic wards, for example, there is no sitting-room for male epileptics, and the patients, the report says, "eat, sleep, and have their fits in one room."

Another improvement, and a very important one, is being pressed by Mayor Strong. It is that the employés of the Charities Department should be "placed under Civil Service rules," which means that they will not be subjected to the wholesale turning out to which American officials generally are liable after every election. By this means it is hoped to obtain suitable permanent officials, appointed by reason of fitness and not for political motives. We think this is absolutely essential to the proper working of the Department.

